# Supplementary material for: Cryo-EM structure of hexameric yeast Lon protease (PIM1) highlights the importance of conserved structural elements
Source: J Biol Chem. 2022 Feb 7;298(3):101694. doi: 10.1016/j.jbc.2022.101694 (PMC8913295; doi:10.1016/j.jbc.2022.101694)
Supplement: Supplementary Table S1 [file mmc1.docx]

**Supplementary Table 1. Cryo-EM data collection, refinement, and validation**

|  | | **EMDB: 25502**  **PDB: 7SXO** |
| --- | --- | --- |
| **Data collection** |  |  |
| Microscope | | Talos Arctica |
| Voltage (keV) | | 200 |
| Detector | | K2 Summit (Counting) |
| Magnification (nominal/calibrated) | | 36,000X / 43,478X |
| Exposure navigation | | Image shift to 16 holes |
| Data acquisition software | | Leginon |
| Total electron exposure (e^-^/Å^2^) | | 50 |
| Exposure rate (e^-^/pixel/sec) | | 6.8 |
| Frame length (ms)  Number of frames per micrograph | | 100  97 |
| Pixel size (Å) | | 1.15 |
| Defocus range (µm) | | -0.7 to -1.5 |
| Micrographs collected (no.) | | 1,394 |
| **Reconstruction** |  |  |
| Image processing package | | CryoSparc |
| Total extracted particles (no.) | | 497,129 |
| Refined particles (no.) | | 111,203 |
| Final particles (no.) | | 111,203 |
| Symmetry imposed | | C1 |
| Resolution (Å) |  |  |
| FSC 0.5 (unmasked / masked) | | 4.4 / 3.5 |
| FSC 0.143 (unmasked / masked) | | 3.8 / 3.2 |
| Resolution range (local) | | 3.2 – 5.0 |
| 3DFSC Sphericity | | 0.975 out of 1 |
| Sharpening B-factor (Å^2^) | | -82.7 |
| **Model Composition** |  |  |
| Protein residues | | 3,613 |
| Ligands | | 12 |
| **Model Refinement** |  |  |
| Refinement package | | Phenix |
| CC (volume / mask) | | 0.75 / 0.74 |
| R.m.s. deviations |  |  |
| Bond lengths | | 0.003 |
| Bond angles (°) | | 0.619 |
| **Validation** |  |  |
| Map-to-model FSC 0.5 | | 3.4 |
| Ramachandran (%) | |  |
| Outliers | | 0.00 |
| Allowed | | 6.96 |
| Favored | | 93.04 |
| MolProbity score | | 1.94 |
| Poor rotamers (%) | | 0.0 |
| Clashscore (all atoms) | | 9.2 |
| C-beta deviations | | 0.00 |
| CaBLAM Outliers (%) | | 4.85 |
| EMRinger Score [35] | | 2.05 |
