# Supplementary material for: Cryo-EM structure of hexameric yeast Lon protease (PIM1) highlights the importance of conserved structural elements
Source: J Biol Chem. 2022 Feb 7;298(3):101694. doi: 10.1016/j.jbc.2022.101694 (PMC8913295; doi:10.1016/j.jbc.2022.101694)
Supplement: Supplementary Figures S1–S4 [file mmc2.docx]

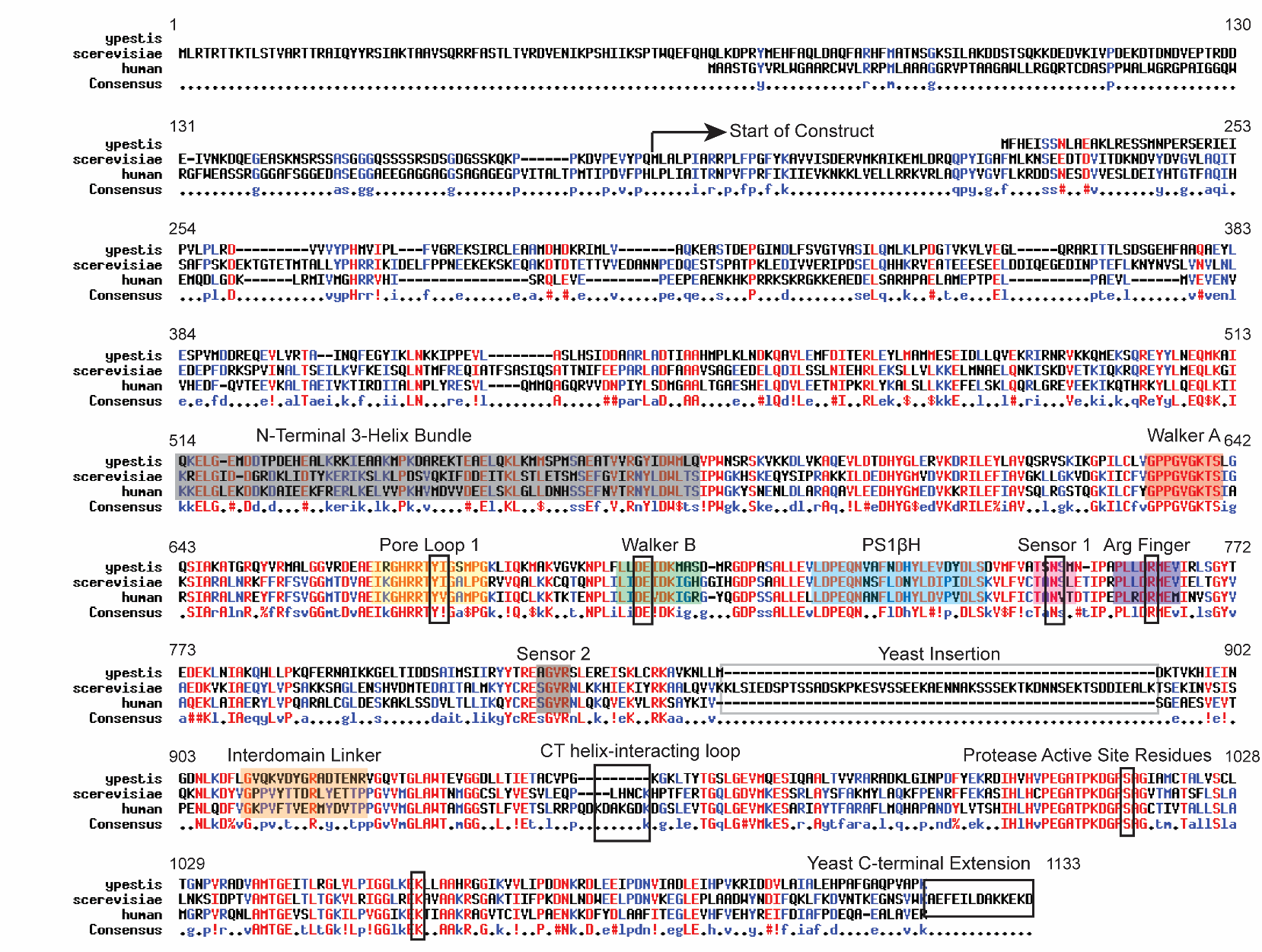


**Supplemental Figure 1. Sequence alignment of *S.cerevisiae* Lon (PIM1), *Yersenia pestis* Lon, and human Lon.** Sequence alignment highlights conserved structural elements: N-terminal 3-helix bundle (gray), Walker A (orange), Pore loop 1 (yellow, aromatic residue Y674 and hydrophobic residue I675 boxed), Walker B (green, nucleotide coordinating acidic residues boxed), Pre-sensor 1 beta-hairpin (light blue), Sensor 1 (pink, N750 boxed), Arginine finger (purple, R762 boxed), Sensor 2 (gray), yeast-specific insertion (gray box), interdomain linker (yellow), CT helix interacting loop (black box), protease active sites (black box, S1015; K1058), Yeast-specific C-terminal extension. Numbering corresponds to PIM1 sequence.


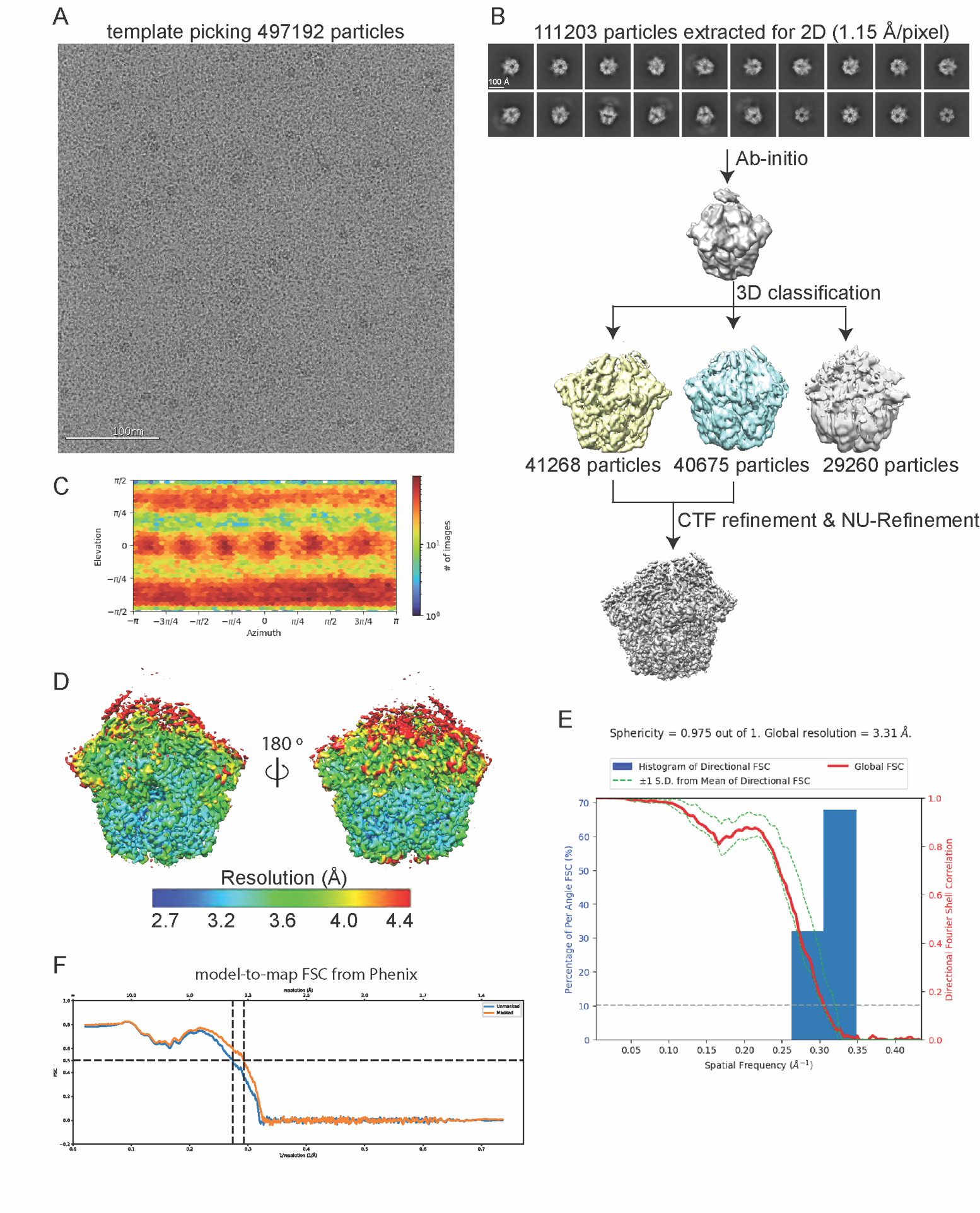
**Supplemental Figure 2. Cryo-EM structure determination of PIM1.** (A) Representative micrograph of cryo-EM data collection, showing high background of what appears to be monomeric PIM1. (B) Workflow of cryo-EM data processing using CryoSparc software [29]. The final 3D reconstruction map was used for model building and refinement. (C) Euler angle distribution plot of the particles used in the final reconstruction. (D) Final reconstruction filtered and colored by local resolution from CryoSparc. (E) 3-Dimensional Fourier Shell Correlation (3DFSC) [36] of the final reconstruction reporting a global resolution of 3.3 Å. (F) Model-to-map FSC plot from Phenix validation.


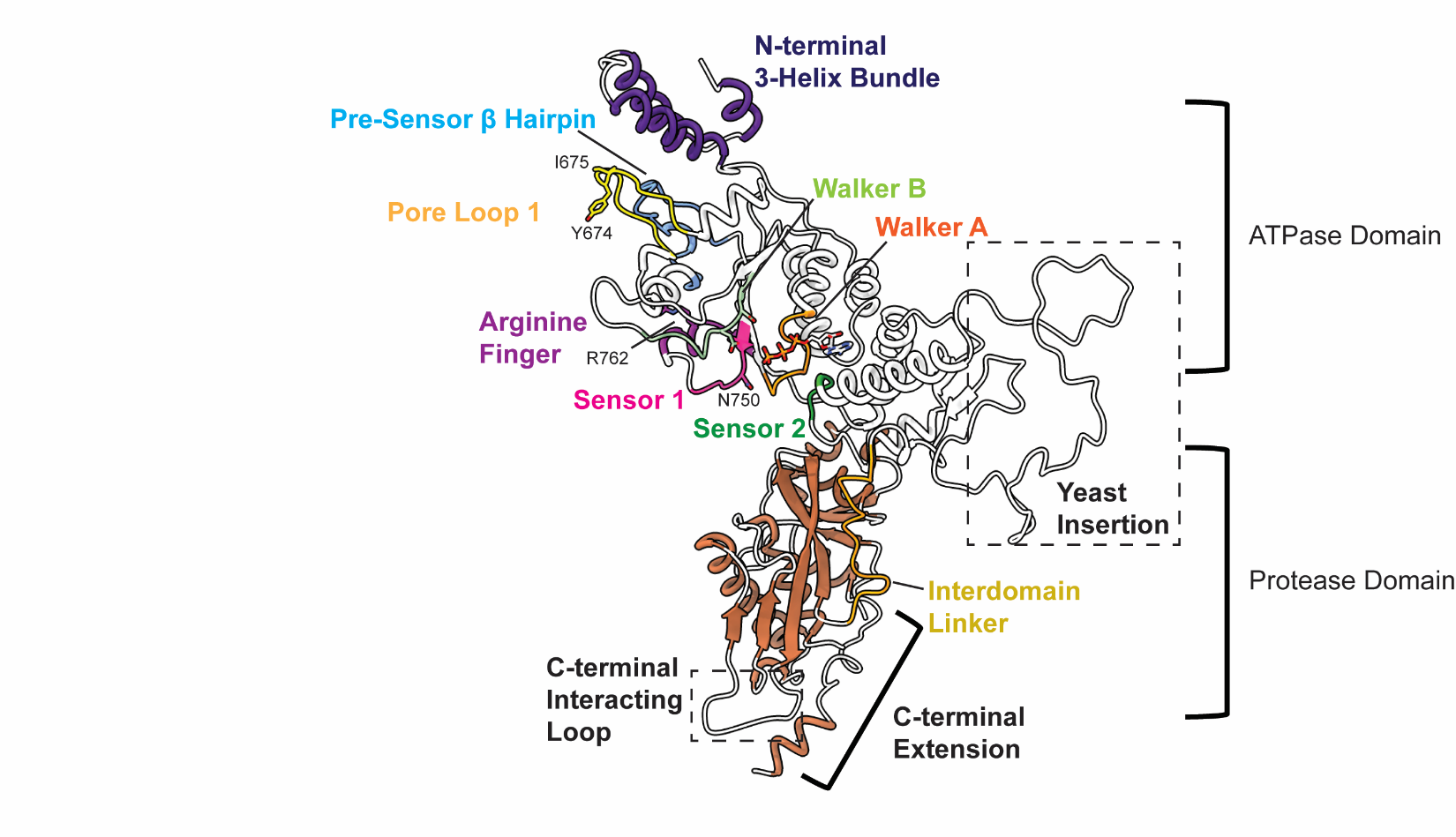


**Supplemental Figure 3. Subunit architecture of substate-translocating PIM1.** PIM1 protomer labeled and colored according to structural domains highlighted in Supplementary Figure 1.


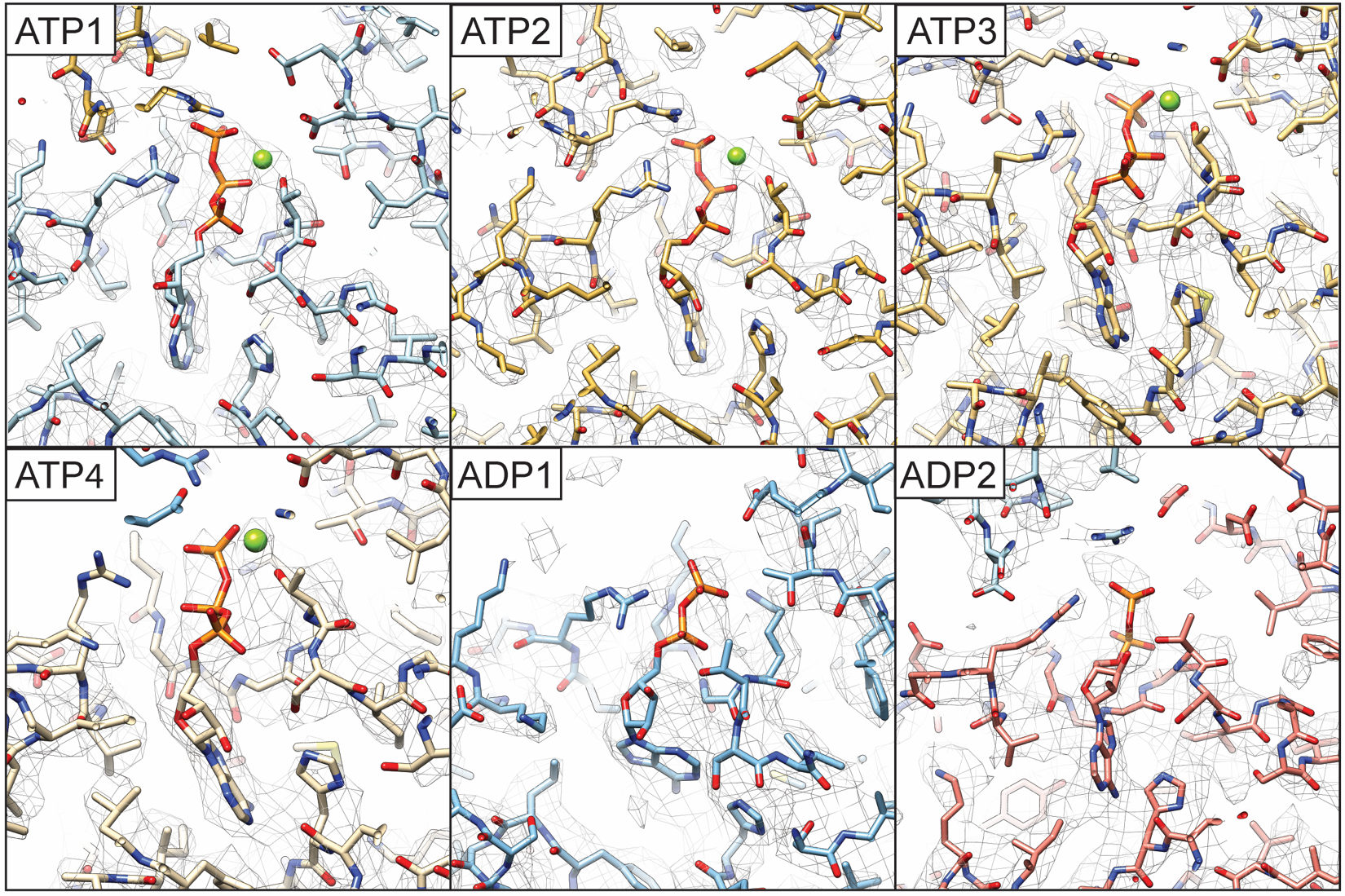


**Supplemental Figure 4. Reconstructed density for nucleotide binding pockets enables assignment of nucleotide states.** Views of the nucleotide binding pocket of all six PIM1 subunits colored according to Figure 1, with atomic model represented as sticks and EM density as a mesh. Green spheres depict Magnesium and coloring of protomers is consistent with that of Figure 1.

References:

29.A. Punjani, J.L. Rubinstein, D.J. Fleet and M.A. Brubaker, cryoSPARC: Algorithms for rapid unsupervised cryo-EM structure determination, Nat. Methods, 14, 2017, 290–296.

35.B.A. Barad, N. Echols, R.Y. Wang, Y. Cheng, F. DiMaio, P.D. Adams and J.S. Fraser, EMRinger: Side chain-directed model and map validation for 3D cryo-electron microscopy, Nat. Methods, 12, 2015, 943–946.

36. Tan, Y. Z., Baldwin, P. R., Davis, J. H., Williamson, J. R., Potter, C. S., Carragher, B., & Lyumkis, D. (2017). Addressing preferred specimen orientation in single-particle cryo-EM through tilting. *Nat Methods, 14*(8), 793-796.
